# CT feature-based nomogram for predicting tumor spread through air spaces in stage IA lung adenocarcinoma

**DOI:** 10.1186/s40644-025-00893-x

**Published:** 2025-06-11

**Authors:** Bin Luo, Han Yang, Ningbo Fan, Pengfei Duan, Zhesheng Wen, Peng Lin

**Affiliations:** Department of Thoracic Surgery, State Key Laboratory of Oncology in South China, Collaborative Innovation Center for Cancer Medicine, Sun Yat-sen University Cancer Center, Guangzhou, 510000 PR China

**Keywords:** Lung adenocarcinoma, Spread through air spaces, Clinicopathologic features, CT features, Nomogram

## Abstract

**Objectives:**

This research aimed to examine the relationships between clinicopathological characteristics and the occurrence of Spread Through Air Spaces (STAS) in patients with stage IA lung adenocarcinoma (LUAD) and to develop a preoperative prediction model.

**Methods:**

Data from 1,375 patients with stage IA LUAD at Sun Yat-sen University Cancer Center were analyzed. Propensity score matching (PSM) was employed to match 141 STAS-positive patients with 282 STAS-negative patients. Both univariate and multivariate logistic regression analyses were performed to determine independent variables among 16 clinicopathological and 13 CT imaging characteristics. A nomogram prediction model was developed and evaluated via receiver operating characteristic (ROC) and decision curve analyses (DCAs).

**Results:**

Multivariate analysis identified several independent risk factors. Irregular nodule shape (OR = 1.817, 95% CI: 1.106–2.986, *p* = 0.018), irregular margin (OR = 2.050, 95% CI: 1.218–3.449, *p* = 0.007), lobulation (OR = 2.235, 95% CI: 1.336–3.739, *p* = 0.002), and vascular convergence (OR = 5.032, 95% CI: 2.050–12.349, *p* < 0.001) were significantly associated with an increased risk of STAS. Compared with a consolidation tumor ratio (CTR) = 0% (reference), a CTR of 75–100% (OR = 7.086, 95% CI: 2.542–19.750, *p* < 0.001) and a CTR = 100% (OR = 11.502, 95% CI: 4.752–27.840, *p* < 0.001) were significantly associated with an increased risk of STAS. The nomogram was developed and internally validated, demonstrating good predictive accuracy (AUC = 0.812, 95% CI: 0.761–0.863) and favorable clinical utility, with a sensitivity of 69.5% and a specificity of 80.2%.

**Conclusion:**

The nomogram reliably predicts STAS preoperatively and may assist in guiding surgical decision-making.

**Supplementary Information:**

The online version contains supplementary material available at 10.1186/s40644-025-00893-x.

## Introduction

Lung cancer exhibits one of the highest incidence and mortality rates globally. According to the global cancer statistics from 2020, lung cancer constitutes approximately 12.4% of all cancer cases and accounts for about 18.7% of all cancer deaths, rendering it the most prevalent and lethal malignant tumor among males [1]. LUAD is the most common histological subtype of non-small cell lung cancer (NSCLC) [2]. Traditionally, lobectomy has been the standard surgical approach for early-stage lung cancer [3]. However, advancements in chest CT screening and imaging technologies over the past two decades have led to the detection of more early-stage tumors, sparking interest in sublobar resection as an alternative for stage IA NSCLC patients [4–8].

In recent years, STAS has been recognized as a distinct invasive pattern in lung cancer. First introduced by Kadota et al. in 2015 [9], STAS was incorporated into the World Health Organization (WHO) classification of lung cancer in the same year. STAS is defined as the spread of tumor cells into alveolar spaces beyond the primary tumor margin within the lung parenchyma [10]. Current studies indicate that STAS is a risk factor for postoperative recurrence in patients with early-stage LUAD [9, 11–17]. Accumulating evidence suggests that STAS-positive patients undergoing sublobar resection have poorer disease-free survival (DFS) and overall survival (OS) compared to those undergoing lobectomy [14, 18–24]. Moreover, a multicenter retrospective study by Chen et al. demonstrated that postoperative adjuvant chemotherapy can improve the prognosis of stage IA LUAD patients with STAS who underwent sublobar resection [25]. These findings underscore the importance of implementing appropriate therapeutic strategies for STAS-positive patients to enhance their prognosis.

The diagnosis of STAS currently relies on adequate postoperative pathological sampling and examination. However, preoperative or intraoperative identification is even more critical for selecting optimal therapeutic strategies. Preoperative STAS detection remains challenging due to the lack of reliable diagnostic tools, and intraoperative frozen section analysis has limited accuracy in identifying STAS [26].

This study aims to explore the risk factors associated with the occurrence of STAS in stage IA LUAD patients treated at our center and to establish an accurate STAS prediction model based on preoperative independent influencing factors, guiding the selection of optimal surgical strategies for these patients.

## Methods

### Study population

We retrospectively analyzed postoperative pathological data from 3,699 patients diagnosed with lung cancer who received surgical treatment at Sun Yat-sen University Cancer Center between December 2020 and October 2022. Patients were categorized into STAS-positive and STAS-negative groups based on postoperative pathological findings.

The inclusion criteria were as follows: (i) histopathological confirmation of LUAD following surgical treatment; (ii) postoperative pathological stage confirmed as stage IA according to the 8th edition of the TNM staging system by the International Association for the Study of Lung Cancer (IASLC); and (iii) availability of complete clinical data.

The exclusion criteria were as follows: (i) lack of preoperative chest CT scans within three months before surgery; (ii) receipt of neoadjuvant treatments (e.g., chemotherapy, radiotherapy, immunotherapy, or targeted therapy); (iii) postoperative pathological diagnosis of carcinoma in situ or minimally invasive adenocarcinoma; (iv) presence of multiple invasive adenocarcinomas in the same lung lobe; (v) history of prior lung surgery; (vi) history of other malignancies.

**Fig. 1:**
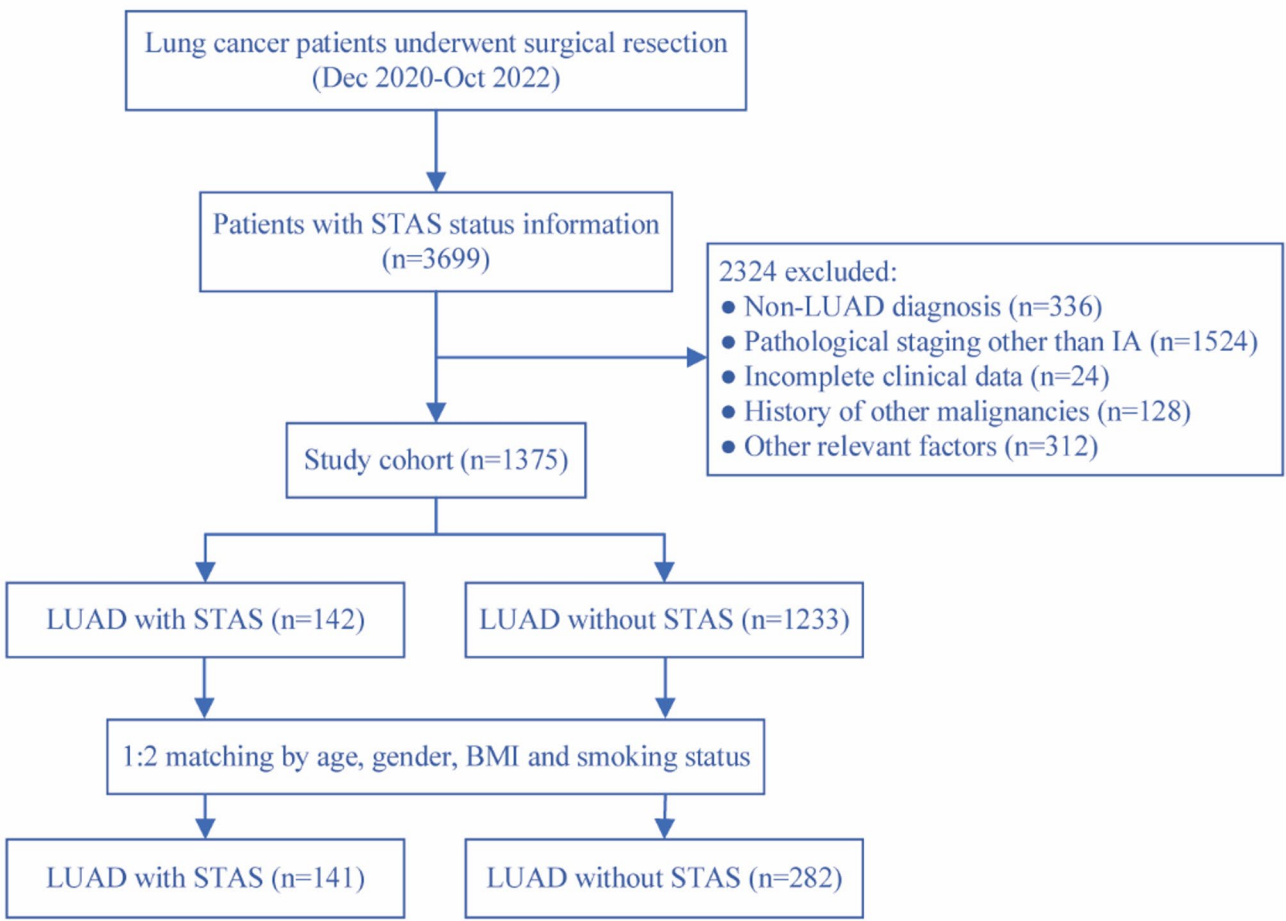
Flow chart for the study population

Given the imbalance in the number of cases between STAS-positive and STAS-negative groups, PSM was applied to reduce confounding bias. The matching variables included demographic factors (age, gender), body mass index (BMI), and smoking status. Matching was performed at ratios of 1:1, 1:2, and 1:3, with a 1:2 matching ratio ultimately selected to optimize the balance between group size and data quality (Supplementary Table 1). A detailed flowchart of the patient selection process is presented in Fig. 1.

### Evaluation of pathological data

**Fig. 2:**
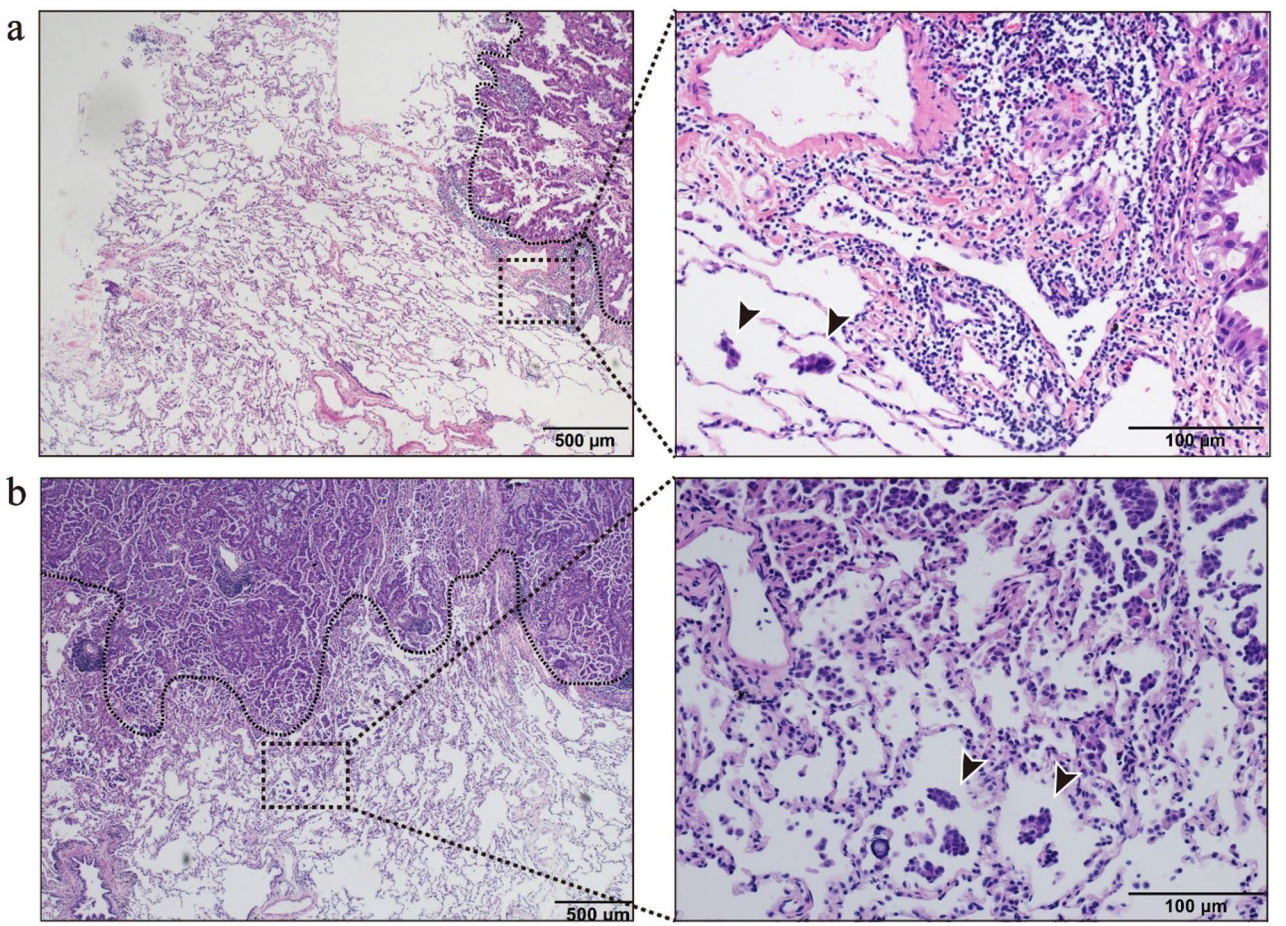
Histopathological features of patients with stage IA LUAD who are positive for STAS. This figure shows the diagnosis of STAS through H&E staining. The tumor that spreads through air spaces is located outside the edge of the primary tumor (black dashed line). **(a)** In a 33-year-old male patient with LUAD, the pathological subtype was mainly acinar; **(b)** In a 38-year-old male patient with LUAD, the pathological subtype was mainly micropapillary

Pathological specimens obtained from lung cancer surgeries were evaluated by two specialized pathologists in accordance with the WHO definition of STAS. Hematoxylin and eosin (H&E) staining was performed on all specimens, with representative findings illustrated in Fig. 2. Discrepancies between the two pathologists were resolved through joint discussions. Histological classification adhered to the criteria jointly proposed by the IASLC, the American Thoracic Society, and the European Respiratory Society. The percentages of histological subtypes were recorded in increments of 5%, with subtypes comprising at least 5% of the tumor considered present. The predominant histological subtype, defined as the subtype with the highest percentage, was utilized for classification and further analysis [27]. Additionally, the degree of tumor differentiation, lymphovascular invasion, and perineural invasion were specifically documented, along with other relevant pathological features.

### Evaluation of CT data

**Fig. 3:**
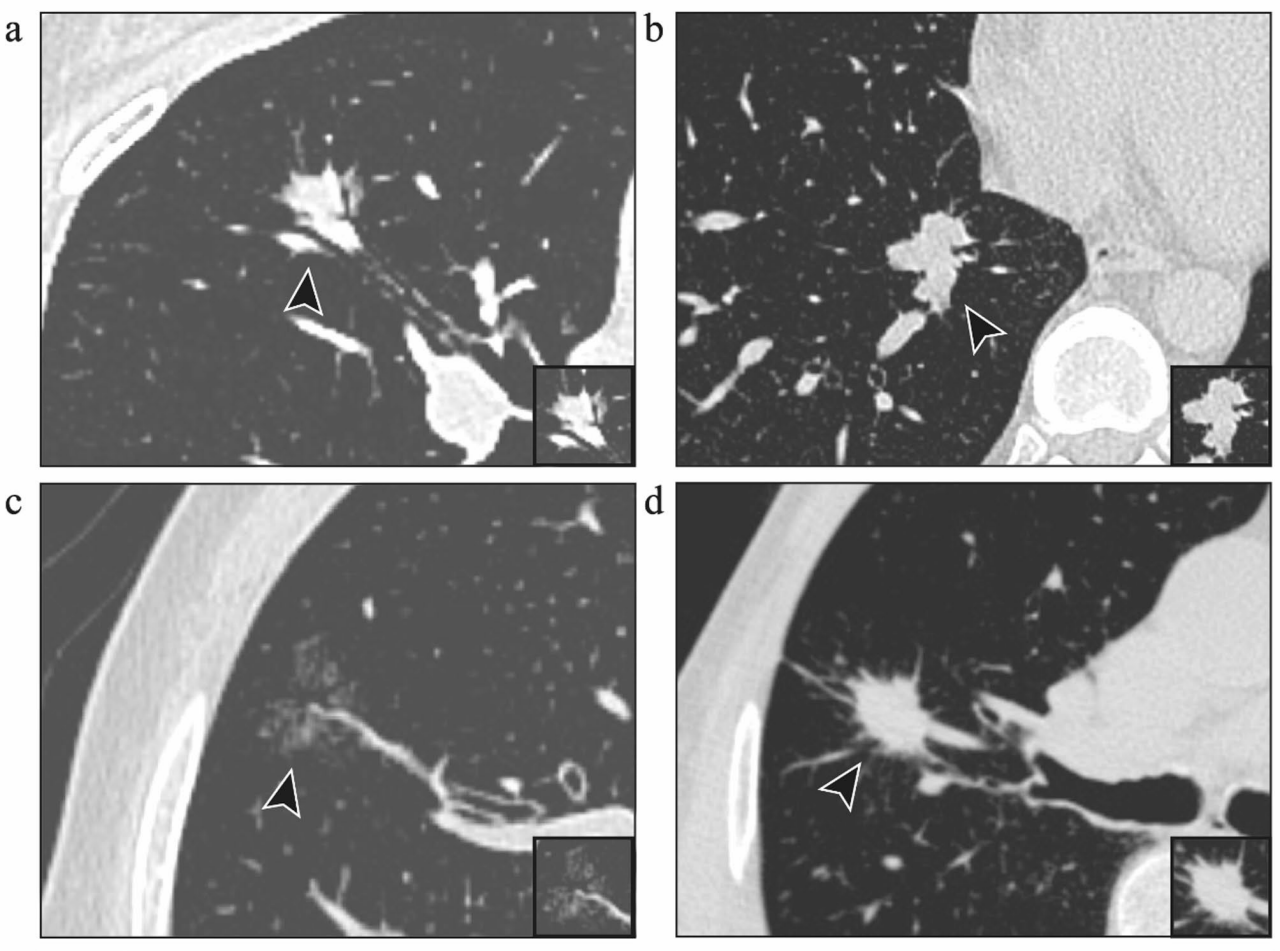
Representative imaging features in patients with stage IA LUAD who are positive for STAS. **(a)** Air bronchogram sign; **(b)** Lobulation sign; **(c)** Vascular convergence sign; **(d)** Spiculation sign

The chest CT images were analyzed independently by two radiologists specializing in lung cancer, including a physician with 2 years of experience and an attending physician with 5 years of experience in diagnostic radiology. Image analysis was performed using the picture archiving and communication system (PACS). To ensure unbiased evaluation, both radiologists were blinded to the postoperative pathological diagnosis, including STAS status. The radiological features assessed included nodule type, size, CTR, lobulation, vascular convergence, spiculation, and other relevant characteristics (Fig. 3). The long- and short-axis diameters of the nodule and its solid component were measured on the largest cross-sectional plane. Discrepancies between the two radiologists were resolved through re-evaluation by a third independent radiologist, a chief physician with 20 years of experience. Among all cases, 7.8% required arbitration.

### Statistical analysis

Statistical analyses were conducted using SPSS (v27.0) and R software (v4.4.0). PSM was performed with the ‘MatchIt’ package in R to balance covariates, including age, gender, BMI, and smoking status, between the two groups. Continuous variables were summarized as mean ± standard deviation when normally distributed or as median (interquartile range) otherwise, with group comparisons conducted using t-tests or Wilcoxon rank-sum tests. Categorical variables were expressed as frequencies and percentages and compared using chi-square or Fisher’s exact tests. Both univariable and multivariable logistic regression analyses were performed to identify significant predictors of STAS. To assess potential multicollinearity among the variables included in the multivariate logistic regression model, we calculated the variance inflation factors (VIFs) in both the training and validation cohorts. The VIF values for all variables were below 1.15 in the training set and below 1.24 in the validation set, both of which are substantially lower than the commonly accepted threshold of 5. Therefore, multicollinearity does not pose a significant concern in our model. The dataset was randomly divided into training and validation sets in a 70:30 ratio using the base functions in R, with a fixed random seed (set.seed(1234)) to ensure reproducibility. A nomogram was developed using the ‘rms’ package in R, and its predictive performance was assessed through ROC curves and calibration curves. Statistical significance was defined as a two-sided *p* value of less than 0.05.

## Results

### Demographic and clinical characteristics

A total of 3,789 patients diagnosed with primary lung cancer were initially screened. After the implementation of the inclusion and exclusion criteria, 1,375 patients with stage IA LUAD were retained for the final analysis, comprising 141 STAS-positive and 1,234 STAS-negative patients. Following PSM, 141 STAS-positive and 282 STAS-negative patients were selected for further analysis (Fig. 1).

The study population consisted of 220 males and 203 females, with a mean age of 57.7 ± 10.9 years. Among the patients in the STAS-positive group, 19.1% (27/141) underwent sublobar resection, which was significantly lower than the 39.7% (112/282) in the STAS-negative group (*P* < 0.001). Univariate analysis indicated no statistically significant differences between the two groups in terms of comorbidities, serum carcinoembryonic antigen (CEA) levels, or family history of lung cancer (*P* > 0.05). However, among patients who underwent postoperative genetic testing, the EGFR mutation status differed significantly between the groups (*P* < 0.05), with wild-type EGFR being more prevalent in the STAS-positive group. A summary of the clinical characteristics of the study population is presented in Table 1.

**Table 1:** Relationships between the STAS and clinicopathological features

Variable	All patients(*n* = 423)	STAS(+)	STAS(-)	*P* Value
**Age(year)**	57.7 ± 10.9	57.7 ± 11.1	57.7 ± 10.9	0.965
**Gender**				0.945
Male	220(52.0)	73(51.8)	147(52.1)	
Female	203(48.0)	68(48.2)	135(47.9)	
**Smoking status**				0.543
Present	150(35.5)	50(64.5)	100(35.5)	
Absent	273(64.5)	91(64.5)	182(35.5)	
**BMI**	23.3 ± 3.2	23.4 ± 3.3	23.3 ± 3.2	0.828
**Family history of lung cancer**				0.543
Present	37(8.7)	14(9.9)	23(8.2)	
Absent	386(91.3)	127(90.1)	259(91.8)	
**Comorbidities**				0.24
Hypertension	91(19.5)	58(20.6)	33(18.9)	
Diabetes	43(9.2)	15(9.4)	28(9.1)	
Heart disease	16(3.4)	9(5.6)	7(2.3)	
Hepatitis	9(1.9)	5(3.1)	4(1.3)	
Others	32(6.9)	12(7.5)	20(6.5)	
**CEA(mg/dL)**				0.306
Normal	343(81.1)	111(78.7)	222(82.3)	
Abnormal	33(7.8)	15(10.6)	18(6.4)	
N/A	47(11.1)	15(10.6)	32(11.3)	
**Surgery**				**<0.001**
Lobectomy	284(67.1)	114(80.9)	170(60.3)	
Sublobar resection	139(32.9)	27(19.1)	112(39.7)	
**pT stage**				**<0.001**
T1a	71(16.8)	10(7.1)	61(21.6)	
T1b	232(54.8)	78(55.3)	154(54.6)	
T1c	120(28.4)	53(37.6)	67(23.8)	
**Tumor differentiation**				**< 0.001**
Well	30(7.1)	3(2.1)	27(9.6)	
Moderate	333(78.7)	94(66.7)	239(84.7)	
poor	60(14.2)	40(31.2)	16(5.7)	
**Histologic subtypes**				
Lepidic predominant	40(9.5)	1(0.7)	39(13.8)	**<0.0001**
Acinar predominant	282(66.6)	92(65.2)	190(67.4)	0.662
Papillary predominant	64(15.1)	26(18.5)	38(13.4)	0.179
Micropapillary predominant	7(1.7)	6(4.3)	1(0.4)	**0.006**_**a**_
Solid predominant	8(1.9)	5(3.5)	3(1.1)	0.077
Others _b_	22(5.2)	11(7.8)	11(3.9)	0.089
**Micropapillary Component**				**0.001**
Present	105(24.8)	80(56.7)	25(8.9)	
Absent	318(75.2)	61(43.3)	257(91.1)	
**Lymphovascular invasion**				**<0.001**
Present	25(5.9)	21(14.9)	4(1.4)	
Absent	398(94.1)	120(85.1)	278(98.6)	
**Perineural invasion**				1.000
Present	2(0.5)	1(0.4)	1(0.7)	
Absent	421(99.5)	281(99.6)	140(99.3)	
**EGFR Mutation**	125(61.0)	38(51.4)	87(66.4)	**0.034**
**ALK Rearrangement**	7(3.6)	5(7.0)	2(1.6)	0.103

EGFR: epidermal growth factor receptor; ALK: anaplastic lymphoma kinase; a: Fisher’s exact test; b: other pathological subtypes, including complex glandular patterns and the mucinous type

### Pathological features

Univariate analysis of pathological characteristics revealed significant differences between the STAS-positive and STAS-negative groups in terms of tumor T stage, degree of differentiation, and predominant pathological subtype. In the STAS-positive group, 56.7% of tumors exhibited a micropapillary structure, and 14.9% demonstrated lymphovascular invasion, both of which were significantly greater than those in the STAS-negative group. Detailed comparisons of pathological characteristics are provided in Table 1.

### Radiological features

**Fig. 4:**
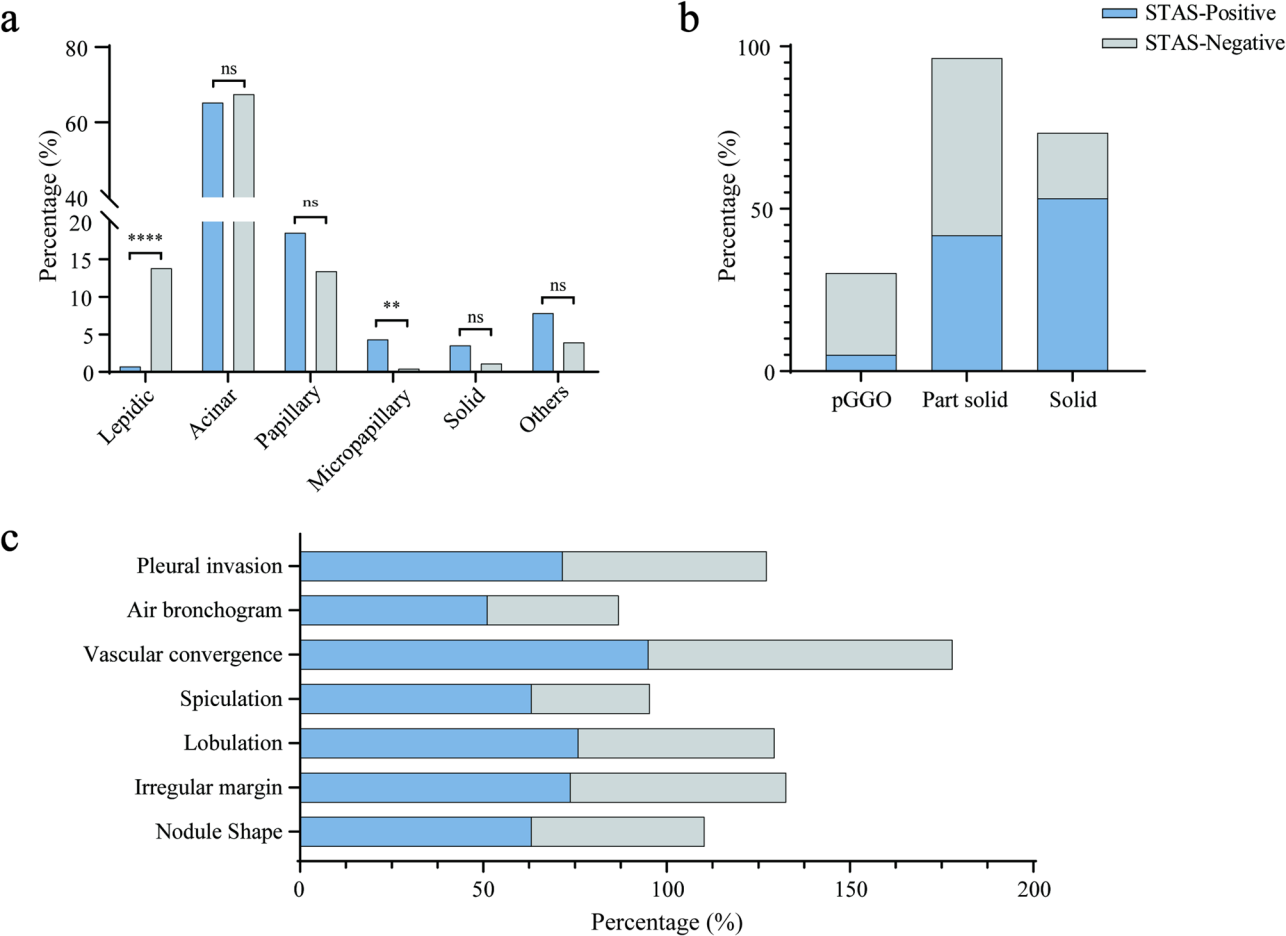
Comparison of STAS-positive and STAS-negative features in lung nodules. **(a)** The clustered column chart compares the predominant pathological subtypes between the STAS-positive and STAS-negative groups. **(b)** The stacked bar chart illustrates the significnat differences in nodule types between the STAS-positive and STAS-negative groups. **(c)** The stacked bar chart shows that STAS is more frequently observed in patients with irregular nodules, irregular margins, lobulation, spiculation, vascular convergence, air bronchogram, and pleural invasion signs. **p < 0.01, **p < 0.0001, ns, *p* > 0.05

Univariate analysis of chest CT features revealed that both the maximum nodule diameter and the maximum diameter of the solid component within the nodule were significantly larger in the STAS-positive group compared to the STAS-negative group (19.2 mm vs. 16.5 mm and 15.0 mm vs. 8.6 mm, respectively; both *P* < 0.001). The CTR was also significantly different between the groups (*P* < 0.001), with STAS positivity increased notably when CTR ≥ 75% and peaked at CTR = 100% (Supplementary Fig. 1). While the right upper lobe was the most common location for nodules (33.8% of cases), no significant difference in nodule location was observed between the groups (*P* > 0.05). However, the type of nodule was significantly different (*P* < 0.001). Radiological features such as irregular nodule shape, irregular margins, lobulation, spiculation, vascular convergence, air bronchogram, and pleural invasion were significantly more prevalent in the STAS-positive group. These findings are summarized in Table 2 and visually illustrated in Fig. 4.

**Table 2:** Relationships between the STAS and CT features

Variable	All patients(*n* = 423)	STAS(+)	STAS(-)	*P* Value
**Maximum tumor diameter (mm)**	17.4 ± 6.3	19.3 ± 5.9	16.5 ± 6.2	**<0.001**_**a**_
**Maximum solid component diameter (mm)**	10.7 ± 8.3	15.1 ± 7.9	8.6 ± 7.6	**<0.001**_**a**_
**CTR (%)**				**<0.001**
0	78(18.4)	7(5.0)	71(25.2)	
0< CTR ≤ 25	26(6.1)	4(2.8)	22(7.8)	
25< CTR ≤ 50	73(17.3)	16(11.3)	57(20.2)	
50< CTR ≤ 75	71(16.8)	18(12.8)	53(18.8)	
75< CTR<100	43(10.2)	21(14.9)	22(7.8)	
100	132(31.2)	75(53.2)	57(20.2)	
**Nodule type**				**<0.001**
pGGO	78(18.4)	7(5.0)	71(25.2)	
Part solid	213(50.4)	59(41.8)	154(54.6)	
Solid	132(31.2)	75(53.2)	57(20.2)	
**Nodule location**				0.163
RUL	143(33.8)	39(27.7)	104(36.9)	
RML	28(6.6)	7(5.0)	21(7.4)	
RLL	89(21.0)	37(26.2)	52(18.4)	
LUL	106(25.1)	39(27.7)	67(23.8)	
LLL	57(13.5)	38(13.5)	19(13.5)	
**Nodule Shape**				**0.002**
Irregular	222(52.5)	89(63.1)	133(47.2)	
Round to oval	201(47.5)	52(36.9)	149(52.8)	
**Irregular margin**				**0.003**
Present	270(63.8)	104(73.8)	166(58.8)	
Absent	153(36.2)	37(26.2)	116(41.1)	
**Lobulation**				**<0.0001**
Present	258(61.0)	107(75.9)	151(53.5)	
Absent	165(39.0)	34(24.1)	131(46.5)	
**Spiculation**				**<0.001**
Present	180(42.6)	89(63.1)	91(32.3)	
Absent	243(57.4)	52(36.9)	191(67.7)	
**Cavitation**				0.418
Present	75(17.7)	28(19.9)	47(16.7)	
Absent	348(82.3)	113(80.1)	235(83.3)	
**Vascular convergence**				**<0.001**
Present	368(87.0)	134(95.0)	234(83.0)	
Absent	55(13)	7(5)	48(17)	
**Air bronchogram**				**0.003**
Present	173(40.9)	72(51.1)	101(35.8)	
Absent	250(59.1)	69(48.9)	181(64.2)	
**Pleural invasion**				**0.002**
Present	258(61.0)	101(71.6)	157(55.7)	
Absent	165(39.0)	40(28.4)	125(44.3)	

CTR: Consolidation tumor ratio; a: Fisher’s exact test

### Multivariate logistic regression analysis

Clinically significant pathological and CT features identified through univariate analysis were further evaluated using multivariate logistic regression analysis. This multivariate analysis revealed several independent risk factors associated with STAS. Among the tumor differentiation subgroups, well-differentiated tumors were significantly correlated with STAS (*p* = 0.009). Lymphovascular invasion (OR = 4.677, 95% CI: 1.371–15.955, *p* = 0.014) and micropapillary structure (OR = 9.08, 95% CI: 5.172–15.938, *p* < 0.001) were also significantly associated with increased risk, whereas lepidic predominant histologic subtypes (OR = 0.069, 95% CI: 0.008–0.575, *p* = 0.013) served as protective factors against STAS (see Table 3). In terms of CT features, nodule shape (OR = 1.817, 95% CI: 1.106–2.986, *p* = 0.018), irregular margin (OR = 2.050, 95% CI: 1.218–3.449, *p* = 0.007), lobulation (OR = 2.235, 95% CI: 1.336–3.739, *p* = 0.002), and vascular convergence (OR = 5.032, 95% CI: 2.050–12.349, *p* < 0.001) were significantly associated with an increased risk. For the CTR, compared with CTR = 0% (reference), both CTR 75–100% (OR = 7.086, 95% CI: 2.542–19.750, *p* < 0.001) and CTR = 100% (OR = 11.502, 95% CI: 4.752–27.840, *p* < 0.001) were significantly associated with an increased risk of STAS. Other radiological features did not show statistical significance (see Table 4).

**Table 3:** Multivariate analysis of pathological features related to STAS

Variable	OR	95%CI	*P* Value
**Tumor differentiation**			
Well	1	─	**0.009**
Moderate	1.542	0.369–6.439	0.553
Poor	4.762	0.996–22.776	0.051
**Lymphovascular invasion**	4.677	1.371–15.955	**0.014**
**Micropapillary structure**	9.08	5.172–15.938	**<0.001**
**Lepidic predominant Histologic subtypes**	0.069	0.008–0.575	**0.013**

**Table 4:** Multivariate analysis of CT features related to STAS

Variable	OR	95%CI	*P* Value
**Nodule Shape**	1.817	1.106–2.986	**0.018**
**Irregular margin**	2.050	1.218–3.449	**0.007**
**Lobulation**	2.235	1.336–3.739	**0.002**
**Vascular convergence**	5.032	2.050-12.349	**<0.001**
**CTR%**			
0	1	─	**<0.001**
0< CTR ≤ 25	1.634	0.412–6.481	0.485
25< CTR ≤ 50	2.118	0.785–5.712	0.138
50< CTR ≤ 75	2.533	0.952–6.738	0.063
75< CTR<100	7.086	2.542–19.750	**<0.001**
100	11.502	4.752–27.840	**<0.001**

### Model development and evaluation

**Fig. 5:**
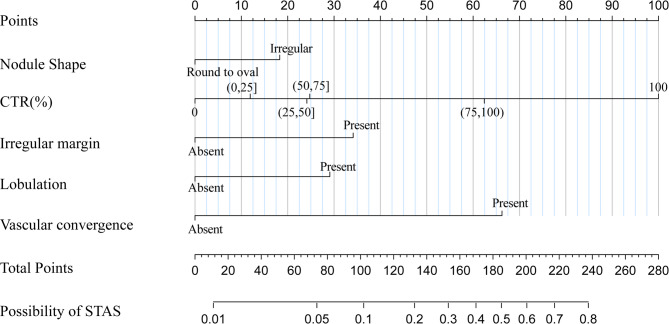
Nomogram model for predicting the occurrence of STAS in stage IA LUAD

Based on multivariate logistic regression analysis, we recognized independent risk factors derived from the features of preoperative CT scans., including nodule shape, irregular margin, lobulation sign, vascular convergence sign, and the CTR, all of which were statistically significant. Using the ‘base’ package in R software, we randomly divided the dataset into two groups at a ratio of 0.7:0.3, employing a fixed random seed to ensure reproducibility. The 0.7 group served as the training set, while the 0.3 group was designated as the internal validation set. Subsequently, the ‘rms’ package in R was utilized to construct a nomogram model based on the significant predictors identified in the training set to predict the occurrence of STAS in stage IA LUAD. As illustrated in Fig. 5, the nomogram assigns points to variables such as nodule shape, the consolidation-to-tumor ratio (CTR%), the presence of an irregular margin, lobulation, and vascular convergence, all identifiable on preoperative CT scans. By summing these points, the total score predicts the probability of STAS positivity. In clinical practice, this nomogram serves as a practical tool to assist surgeons in tailoring their decision-making. Patients with higher predicted probabilities of STAS positivity, based on preoperative CT features, may benefit from more aggressive surgical strategies, such as lobectomy instead of sublobar resection, to mitigate the risk of recurrence. Its simplicity and user-friendly design also make it suitable for integration into routine clinical workflows, facilitating personalized treatment planning and effective communication of risk with patients.

**Fig. 6:**
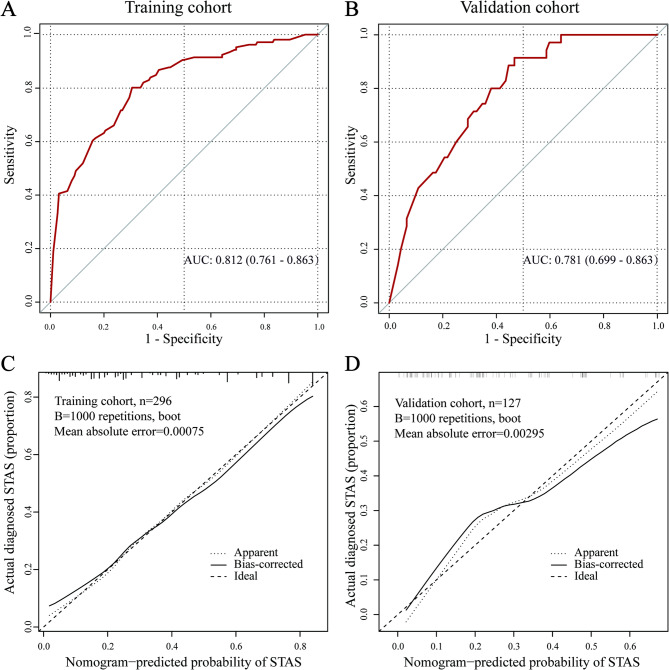
Performance evaluation of the nomogram model in the training set and internal validation set. ROC curves for the nomogram model for predicting stage IA LUAD STAS in the training set **(a)** and validation set **(b)**. The calibration curves for evaluating the nomogram model in the training set **(c)** and validation set **(d)**

**Fig. 7:**
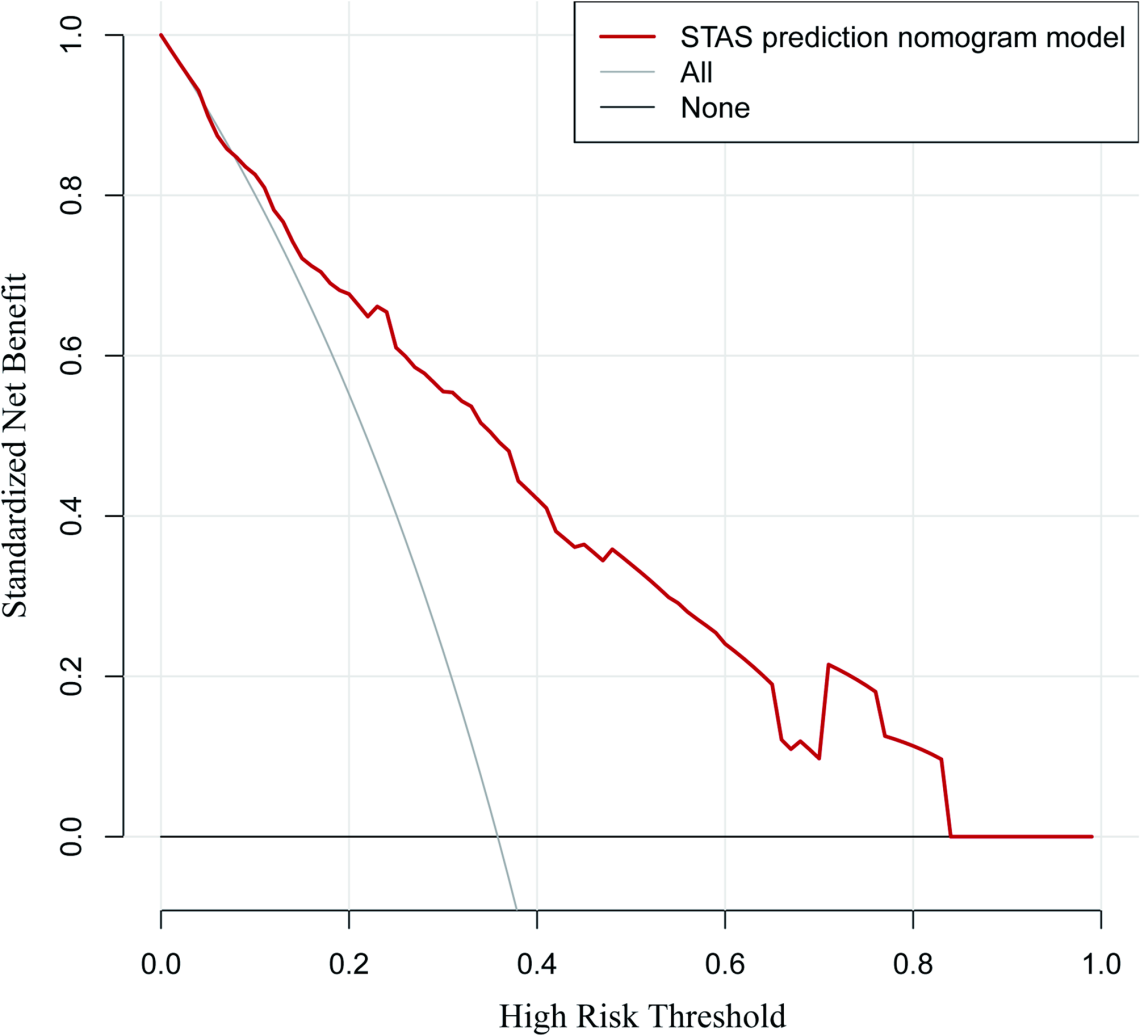
Decision curve analysis of the line chart model

The nomogram demonstrated high predictive accuracy in the training set, achieving an AUC of 0.812 (95% CI: 0.761–0.863). Internal validation further corroborated its robustness, yielding an AUC of 0.781 (95% CI: 0.699–0.863). These AUC values, as shown in Fig. 6a and b, suggest a strong discriminatory ability of the model in predicting the occurrence of STAS. The optimal cut-off value for the training cohort was 33.9%, with a sensitivity of 69.5%, a specificity of 80.2%, and a Youden’s Index of 0.497. For the validation cohort, the optimal cut-off value was 20.1%, with a sensitivity of 91.4%, a specificity of 53.3%, and a Youden’s Index of 0.447. The bootstrap method was employed for internal validation, and the calibration curve (Fig. 6c and d) demonstrates good alignment between predicted and actual probabilities, confirming the model’s accuracy. Furthermore, the decision curve analysis (DCA) illustrated in Fig. 7 indicates that the nomogram model provides a higher clinical net benefit across the risk threshold range of 0.1 to 0.8, underscoring its potential value in clinical practice.

## Discussion

This study identified key clinicopathological factors associated with STAS in stage IA LUAD, including tumor differentiation, micropapillary structure, lymphovascular invasion, and lepidic predominant subtype. STAS was more frequent in tumors with moderate to poor differentiation, and the micropapillary structure was significantly more prevalent in STAS-positive patients, consistent with previous studies [11, 24, 28–33]. Lymphovascular invasion also showed a significant correlation with STAS, highlighting its role in tumor spread [9, 13, 15]. A meta-analysis further supported that STAS is common in micropapillary subtypes but rare in lepidic subtypes, aligning with our findings [34]. In terms of molecular features, some studies have reported a significant correlation between STAS positivity and wild-type EGFR [11, 12]. However, Toyokawa et al. (2018) found no such association with EGFR mutations [16]. In our cohort, STAS-positive patients predominantly exhibited wild-type EGFR (*P* < 0.05), which is consistent with its association with poor prognosis in LUAD [35]. Although 71.4% of patients with ALK rearrangements were STAS-positive, the difference was not statistically significant, likely due to the limited sample size. Additionally, while Shiono et al. reported higher CEA levels in STAS-positive patients [36], our findings did not show a significant difference. Larger cohorts are needed to validate these associations.

While STAS is traditionally confirmed through postoperative pathological examination, numerous retrospective studies have explored its correlation with preoperative CT features, aiming to predict its occurrence [17, 37–40]. Given that STAS is a microscopic phenomenon beyond the spatial resolution of current CT technology, it is suggested that indirect indicators should be utilized for its prediction [17]. Studies by Toyokawa et al. and Kim et al. identified significant associations between STAS and CT features such as maximum nodule diameter, solid component proportion, and CTR, highlighting their predictive potential [16, 17, 41]. In our cohort of 423 patients with stage IA LUAD, STAS was significantly associated with the maximum diameters of both the nodule and its solid component, as well as the CTR. Notably, CTR emerged as an independent risk factor for predicting STAS occurrence, with higher CTR values correlating with an increased likelihood of STAS. Consequently, CTR, which is readily measurable by CT, may facilitate the selection of the optimal surgical strategy.

Regarding nodule type, Kim et al. reported no STAS in pure ground-glass nodules (GGNs) [17]. However, other studies have reported the presence of STAS in pure GGNs, albeit with a relatively low incidence [37, 41, 42]. In our study, 5% (7/141) of the STAS-positive patients exhibited pure GGNs, and 14.1% (20/141) had nodules where the ground-glass component predominated. These findings suggest that the choice between sublobar resection and lobectomy should not be based solely on the presence of pure GGNs but should also incorporate a comprehensive evaluation of imaging features. Consistent with Shiono et al., we found that pure solid nodules had a significantly higher STAS positivity rate compared to subsolid nodules [36]. Additionally, several CT features, such as irregular margin, lobulation, spiculation, vascular convergence, air bronchogram, and pleural invasion, were frequently observed in STAS-positive patients [38, 41, 43, 44]. Similar to these studies, our research confirms that irregular nodule shape, irregular margin, lobulation, spiculation, vascular convergence, air bronchogram, and pleural invasion are commonly seen in CT images of stage IA LUAD patients with STAS. Among these, irregular nodule shape, irregular margin, lobulation sign, and vascular convergence sign were identified as independent risk factors for STAS occurrence in stage IA LUAD. These findings highlight the utility of preoperative chest CT imaging in predicting STAS and optimizing surgical planning for stage IA LUAD patients.

The nomogram developed in this study demonstrated good predictive performance, with an AUC of **0.812** in the training cohort, indicating a notable discriminatory ability for predicting the occurrence of STAS. In recent years, several studies have explored the relationships between CT-based radiomics features and STAS in lung adenocarcinomas [45–48]. These radiomics-based nomograms have been developed to enhance diagnostic accuracy for STAS, with reported AUCs ranging from 0.630 to 0.907. However, the practical application of these tools is partially constrained by the intricate technical requirements of radiomics. Other investigations have leveraged clinical characteristics to estimate the risk of STAS [49–51]; For instance, Wang et al. established a nomogram that achieved AUCs of 0.860 in the training group and 0.919 in the validation group [50]. Their model incorporated postoperative pathological data, such as Ki-67 and PD-L1 expression, which may have contributed to its enhanced predictive accuracy for STAS. In contrast, Chen et al. developed a nomogram based on the percentage of the solid component and lobulation, achieving a C-index of 0.803, which is comparable to the diagnostic performance of our model [49]. Similarly, Yang et al. constructed a nomogram utilizing preoperative CEA levels and CT features, achieving an AUC of 0.835 [51]. Their model demonstrated a sensitivity and specificity of 83.6% and 78.2%, respectively, which are slightly higher than those of our model. This discrepancy may be attributed to their use of larger cohorts, which likely improved statistical power and reduced potential biases. Overall, while the nomogram developed in our study demonstrates relatively favorable predictive performance and potential clinical utility, future research should focus on refining its accuracy and generalizability. This can be achieved by incorporating a broader array of biomarkers and utilizing larger, multi-center cohorts to enhance statistical power and mitigate potential biases.

The clinical significance of our findings is noteworthy; however, several limitations should be acknowledged. First, as a retrospective cohort study focusing exclusively on surgically treated stage IA LUAD patients, there is an inherent risk of selection bias, which may have influenced the representativeness of the sample population. Second, the relatively short postoperative follow-up period limited the evaluation of long-term outcomes, such as disease recurrence and overall prognosis. Future studies with longer follow-up durations are necessary to further elucidate the prognostic impact of STAS. Third, the single-center design restricts the generalizability of our results, while the absence of external validation further limits their robustness. Multicenter studies with diverse patient populations and external validation using independent cohorts are crucial to improve the reliability and applicability of our findings.

## Conclusion

In summary, this study analyzed the clinicopathologic and CT features associated with STAS in patients with stage IA LUAD. We identified lower tumor differentiation, non-lepidic predominant subtypes, micropapillary structure, and lymphovascular invasion as significant clinicopathologic risk factors for STAS. Additionally, CT features such as solid components, irregular shape, irregular margin, lobulation, and vascular convergence were significant predictors of STAS. On the basis of these five CT features, we developed a nomogram that demonstrates promising predictive accuracy and has the potential to guide clinical decision-making. This model may serve as a useful tool for preoperative STAS risk assessment and could assist thoracic surgeons in tailoring surgical strategies for patients with stage IA LUAD.

## Electronic supplementary material

Below is the link to the electronic supplementary material.

Supplementary Material 1

Supplementary Material 2

## Data Availability

No datasets were generated or analysed during the current study.

## Abbreviations

LUAD: Lung Adenocarcinoma
STAS: Spread through Air Spaces
PSM: Propensity Score Matching
NSCLC: Non-small Cell Lung Cancer
CT: Computed Tomography
ROC: Receiver Operating Characteristic
AUC: Area Under the Curve
DCA: Decision Curve Analysis
CI: Confidence Interval
DFS: Disease-Free Survival
OS: Overall Survival
IASLC: International Association for the Study of Lung Cancer
PACS: Picture Archiving and Communication System
BMI: Body Mass Index
CTR: Consolidation Tumor Ratio
CEA: Carcinoembryonic Antigen
GGN: Ground-Glass Nodules

## References

[1] Bray F, Laversanne M, Sung H, Ferlay J, Siegel RL, Soerjomataram I, et al. Global cancer statistics 2022: GLOBOCAN estimates of incidence and mortality worldwide for 36 cancers in 185 countries. CA Cancer J Clin. 2024;74(3):229–63. 38572751 10.3322/caac.21834

[2] Zhang Y, Vaccarella S, Morgan E, Li M, Etxeberria J, Chokunonga E, et al. Global variations in lung cancer incidence by histological subtype in 2020: a population-based study. Lancet Oncol. 2023;24(11):1206–18. 37837979 10.1016/S1470-2045(23)00444-8

[3] Ginsberg RJ, Rubinstein LV. Randomized trial of lobectomy versus limited resection for T1 N0 non-small cell lung cancer. Lung Cancer study group. Ann Thorac Surg. 1995;60(3):615–22. discussion 622–623. 7677489 10.1016/0003-4975(95)00537-u

[4] Onaitis MW, Furnary AP, Kosinski AS, Feng L, Boffa D, Tong BC, et al. Equivalent survival between lobectomy and segmentectomy for clinical stage IA lung Cancer. Ann Thorac Surg. 2020;110(6):1882–91. 32119855 10.1016/j.athoracsur.2020.01.020

[5] Landreneau RJ, Normolle DP, Christie NA, Awais O, Wizorek JJ, Abbas G, et al. Recurrence and survival outcomes after anatomic segmentectomy versus lobectomy for clinical stage I non-small-cell lung cancer: a propensity-matched analysis. J Clin Oncol. 2014;32(23):2449–55. 24982447 10.1200/JCO.2013.50.8762 PMC4121502

[6] Altorki NK, Yip R, Hanaoka T, Bauer T, Aye R, Kohman L, et al. Sublobar resection is equivalent to lobectomy for clinical stage 1A lung cancer in solid nodules. J Thorac Cardiovasc Surg. 2014;147(2):754–62. Discussion 762–764. 24280722 10.1016/j.jtcvs.2013.09.065

[7] Saji H, Okada M, Tsuboi M, Nakajima R, Suzuki K, Aokage K, et al. Segmentectomy versus lobectomy in small-sized peripheral non-small-cell lung cancer (JCOG0802/WJOG4607L): a multicentre, open-label, phase 3, randomised, controlled, non-inferiority trial. Lancet. 2022;399(10335):1607–17. 35461558 10.1016/S0140-6736(21)02333-3

[8] Altorki N, Wang X, Kozono D, Watt C, Landrenau R, Wigle D, et al. Lobar or sublobar resection for peripheral stage IA Non-Small-Cell lung Cancer. N Engl J Med. 2023;388(6):489–98. 36780674 10.1056/NEJMoa2212083 PMC10036605

[9] Kadota K, Nitadori JI, Sima CS, Ujiie H, Rizk NP, Jones DR, et al. Tumor spread through air spaces is an important pattern of invasion and impacts the frequency and location of recurrences after limited resection for small stage I lung adenocarcinomas. J Thorac Oncol. 2015;10(5):806–14. 25629637 10.1097/JTO.0000000000000486 PMC4500042

[10] Travis WD, Brambilla E, Nicholson AG, Yatabe Y, Austin JHM, Beasley MB, et al. The 2015 world health organization classification of lung tumors: impact of genetic, clinical and radiologic advances since the 2004 classification. J Thorac Oncol: Off Publ Int Assoc Study Lung Cancer. 2015;10(9):1243–60. 10.1097/JTO.0000000000000630 26291008

[11] Onozato ML, Kovach AE, Yeap BY, Morales-Oyarvide V, Klepeis VE, Tammireddy S, et al. Tumor Islands in resected early-stage lung adenocarcinomas are associated with unique clinicopathologic and molecular characteristics and worse prognosis. Am J Surg Pathol. 2013;37(2):287–94. 23095504 10.1097/PAS.0b013e31826885fb PMC3545070

[12] Warth A, Muley T, Kossakowski CA, Goeppert B, Schirmacher P, Dienemann H, et al. Prognostic impact of Intra-alveolar tumor spread in pulmonary adenocarcinoma. Am J Surg Pathol. 2015;39(6):793–801. 25723114 10.1097/PAS.0000000000000409

[13] Morimoto J, Nakajima T, Suzuki H, Nagato K, Iwata T, Yoshida S, et al. Impact of free tumor clusters on prognosis after resection of pulmonary adenocarcinoma. J Thorac Cardiovasc Surg. 2016;152(1):64–e721. 27343907 10.1016/j.jtcvs.2016.03.088

[14] Dai C, Xie H, Su H, She Y, Zhu E, Fan Z, et al. Tumor spread through air spaces affects the recurrence and overall survival in patients with lung adenocarcinoma > 2 to 3 cm. J Thorac Oncol. 2017;12(7):1052–60. 28389373 10.1016/j.jtho.2017.03.020

[15] Uruga H, Fujii T, Fujimori S, Kohno T, Kishi K. Semiquantitative assessment of tumor spread through air spaces (STAS) in Early-Stage lung adenocarcinomas. J Thorac Oncol. 2017;12(7):1046–51. 28363628 10.1016/j.jtho.2017.03.019

[16] Toyokawa G, Yamada Y, Tagawa T, Kozuma Y, Matsubara T, Haratake N, et al. Significance of spread through air spaces in resected pathological stage I lung adenocarcinoma. Ann Thorac Surg. 2018;105(6):1655–63. 29453963 10.1016/j.athoracsur.2018.01.037

[17] Kim SK, Kim TJ, Chung MJ, Kim TS, Lee KS, Zo JI, et al. Lung adenocarcinoma: CT features associated with spread through air spaces. Radiology. 2018;289(3):831–40. 30179108 10.1148/radiol.2018180431

[18] Eguchi T, Kameda K, Lu S, Bott MJ, Tan KS, Montecalvo J, et al. Lobectomy is associated with better outcomes than sublobar resection in spread through air spaces (STAS)-Positive T1 lung adenocarcinoma: A propensity Score-Matched analysis. J Thorac Oncol. 2019;14(1):87–98. 30244070 10.1016/j.jtho.2018.09.005 PMC6309668

[19] Kadota K, Kushida Y, Kagawa S, Ishikawa R, Ibuki E, Inoue K, et al. Limited resection is associated with a higher risk of locoregional recurrence than lobectomy in stage I lung adenocarcinoma with tumor spread through air spaces. Am J Surg Pathol. 2019;43(8):1033–41. 31107717 10.1097/PAS.0000000000001285

[20] Ren Y, Xie H, Dai C, She Y, Su H, Xie D, et al. Prognostic impact of tumor spread through air spaces in sublobar resection for 1A lung adenocarcinoma patients. Ann Surg Oncol. 2019;26(6):1901–8. 30887374 10.1245/s10434-019-07296-w

[21] Shiono S, Endo M, Suzuki K, Yarimizu K, Hayasaka K, Yanagawa N. Spread through air spaces is a prognostic factor in sublobar resection of Non-Small cell lung Cancer. Ann Thorac Surg. 2018;106(2):354–60. 29625101 10.1016/j.athoracsur.2018.02.076

[22] Shiono S, Endo M, Suzuki K, Hayasaka K, Yanagawa N. Spread through air spaces in lung cancer patients is a risk factor for pulmonary metastasis after surgery. J Thorac Dis. 2019;11(1):177–87. 30863587 10.21037/jtd.2018.12.21 PMC6384349

[23] Bains S, Eguchi T, Warth A, Yeh YC, Nitadori JI, Woo KM, et al. Procedure-Specific risk prediction for recurrence in patients undergoing lobectomy or sublobar resection for small (≤ 2 cm) lung adenocarcinoma: an international cohort analysis. J Thorac Oncol. 2019;14(1):72–86. 30253972 10.1016/j.jtho.2018.09.008 PMC6309652

[24] Masai K, Sakurai H, Sukeda A, Suzuki S, Asakura K, Nakagawa K, et al. Prognostic impact of margin distance and tumor spread through air spaces in limited resection for primary lung Cancer. J Thorac Oncol. 2017;12(12):1788–97. 28882585 10.1016/j.jtho.2017.08.015

[25] Chen D, Wang X, Zhang F, Han R, Ding Q, Xu X, et al. Could tumor spread through air spaces benefit from adjuvant chemotherapy in stage I lung adenocarcinoma? A multi-institutional study. Ther Adv Med Oncol. 2020;12:1758835920978147. 33403018 10.1177/1758835920978147 PMC7739212

[26] Mino-Kenudson M. Significance of tumor spread through air spaces (STAS) in lung cancer from the pathologist perspective. Transl Lung Cancer Res. 2020;9(3):847–59. 32676351 10.21037/tlcr.2020.01.06 PMC7354155

[27] Travis WD, Brambilla E, Noguchi M, Nicholson AG, Geisinger KR, Yatabe Y, et al. International association for the study of lung cancer/american thoracic society/european respiratory society international multidisciplinary classification of lung adenocarcinoma. J Thorac Oncol. 2011;6(2):244–85. 21252716 10.1097/JTO.0b013e318206a221 PMC4513953

[28] Morales-Oyarvide V, Mino-Kenudson M. Tumor Islands and spread through air spaces: distinct patterns of invasion in lung adenocarcinoma. Pathol Int. 2016;66(1):1–7. 26642845 10.1111/pin.12368

[29] Miyoshi T, Satoh Y, Okumura S, Nakagawa K, Shirakusa T, Tsuchiya E, et al. Early-stage lung adenocarcinomas with a micropapillary pattern, a distinct pathologic marker for a significantly poor prognosis. Am J Surg Pathol. 2003;27(1):101–9. 12502932 10.1097/00000478-200301000-00011

[30] Makimoto Y, Nabeshima K, Iwasaki H, Miyoshi T, Enatsu S, Shiraishi T, et al. Micropapillary pattern: a distinct pathological marker to subclassify tumours with a significantly poor prognosis within small peripheral lung adenocarcinoma (=20 mm) with mixed Bronchioloalveolar and invasive subtypes (Noguchi’s type C tumours)</at. Histopathology. 2005;46(6):677–84. 15910599 10.1111/j.1365-2559.2005.02126.x

[31] Nitadori Jichi, Bograd AJ, Kadota K, Sima CS, Rizk NP, Morales EA, et al. Impact of micropapillary histologic subtype in selecting limited resection vs lobectomy for lung adenocarcinoma of 2 cm or smaller. J Natl Cancer Inst. 2013;105(16):1212–20. 23926067 10.1093/jnci/djt166 PMC3748005

[32] Yi E, Bae MK, Cho S, Chung JH, Jheon S, Kim K. Pathological prognostic factors of recurrence in early stage lung adenocarcinoma. ANZ J Surg. 2018;88(4):327–31. 28702948 10.1111/ans.14033

[33] Shih AR, Mino-Kenudson M. Updates on spread through air spaces (STAS) in lung cancer. Histopathology. 2020;77(2):173–80. 31943337 10.1111/his.14062

[34] Pyo JS, Kim NY. Clinicopathological impact of the spread through air space in Non-Small cell lung cancer: A Meta-Analysis. Diagnostics (Basel). 2022;12(5):1112. 35626268 10.3390/diagnostics12051112 PMC9139777

[35] Yoon HY, Ryu JS, Sim YS, Kim D, Lee SY, Choi J, et al. Clinical significance of EGFR mutation types in lung adenocarcinoma: A multi-centre Korean study. PLoS ONE. 2020;15(2):e0228925. 32053675 10.1371/journal.pone.0228925 PMC7018076

[36] Shiono S, Yanagawa N. Spread through air spaces is a predictive factor of recurrence and a prognostic factor in stage I lung adenocarcinoma. Interact Cardiovasc Thorac Surg. 2016;23(4):567–72. 27354463 10.1093/icvts/ivw211

[37] Zhang Z, Liu Z, Feng H, Xiao F, Shao W, Liang C, et al. Predictive value of radiological features on spread through air space in stage cIA lung adenocarcinoma. J Thorac Dis. 2020;12(11):6494–504. 33282351 10.21037/jtd-20-1820 PMC7711360

[38] Toyokawa G, Yamada Y, Tagawa T, Kamitani T, Yamasaki Y, Shimokawa M, et al. Computed tomography features of resected lung adenocarcinomas with spread through air spaces. J Thorac Cardiovasc Surg. 2018;156(4):1670–e16764. 29961590 10.1016/j.jtcvs.2018.04.126

[39] Ding Y, Chen Y, Wen H, Li J, Chen J, Xu M, et al. Pretreatment prediction of tumour spread through air spaces in clinical stage I non-small-cell lung cancer. Eur J Cardiothorac Surg. 2022;62(3):ezac248. 35385066 10.1093/ejcts/ezac248 PMC9422756

[40] Li C, Jiang C, Gong J, Wu X, Luo Y, Sun G. A CT-based logistic regression model to predict spread through air space in lung adenocarcinoma. Quant Imaging Med Surg. 2020;10(10):1984–93. 33014730 10.21037/qims-20-724 PMC7495322

[41] de Margerie-Mellon C, Onken A, Heidinger BH, VanderLaan PA, Bankier AA. CT manifestations of tumor spread through airspaces in pulmonary adenocarcinomas presenting as subsolid nodules. J Thorac Imaging. 2018;33(6):402–8. 30067571 10.1097/RTI.0000000000000344

[42] Zhong Y, Xu Y, Deng J, Wang T, Sun X, Chen D, et al. Prognostic impact of tumour spread through air space in radiological subsolid and pure solid lung adenocarcinoma. Eur J Cardiothorac Surg. 2021;59(3):624–32. 33188689 10.1093/ejcts/ezaa361

[43] Qi L, Xue K, Cai Y, Lu J, Li X, Li M. Predictors of CT morphologic features to identify spread through air spaces preoperatively in Small-Sized lung adenocarcinoma. Front Oncol. 2020;10:548430. 33505903 10.3389/fonc.2020.548430 PMC7831277

[44] Gu Y, Zheng B, Zhao T, Fan Y. Computed tomography features and tumor spread through air spaces in lung adenocarcinoma: A Meta-analysis. J Thorac Imaging. 2023;38(2):W19–29. 36583661 10.1097/RTI.0000000000000693 PMC9936977

[45] Chen D, She Y, Wang T, Xie H, Li J, Jiang G, et al. Radiomics-based prediction for tumour spread through air spaces in stage I lung adenocarcinoma using machine learning. Eur J Cardio-thorac Surg: Off J Eur Assoc Cardio-thorac Surg. 2020;58(1):51–8. 10.1093/ejcts/ezaa011 32011674

[46] Jiang C, Luo Y, Yuan J, You S, Chen Z, Wu M, et al. CT-based radiomics and machine learning to predict spread through air space in lung adenocarcinoma. Eur Radio. 2020;30(7):4050–7. 10.1007/s00330-020-06694-z 32112116

[47] Qi L, Li X, He L, Cheng G, Cai Y, Xue K, et al. Comparison of diagnostic performance of spread through airspaces of lung adenocarcinoma based on morphological analysis and perinodular and intranodular radiomic features on chest CT images. Front Oncol. 2021;11:654413. 34249691 10.3389/fonc.2021.654413 PMC8268002

[48] Han X, Fan J, Zheng Y, Ding C, Zhang X, Zhang K, et al. The value of CT-based radiomics for predicting spread through air spaces in stage IA lung adenocarcinoma. Front Oncol. 2022;12:757389. 35880159 10.3389/fonc.2022.757389 PMC9307661

[49] Chen Y, Jiang C, Kang W, Gong J, Luo D, You S, et al. Development and validation of a CT-based nomogram to predict spread through air space (STAS) in peripheral stage IA lung adenocarcinoma. Jpn J Radiol. 2022;40(6):586–94. 35079955 10.1007/s11604-021-01240-3

[50] Wang J, Yao Y, Tang D, Gao W. An individualized nomogram for predicting and validating spread through air space (STAS) in surgically resected lung adenocarcinoma: a single center retrospective analysis. J Cardiothorac Surg. 2023;18(1):337. 37990253 10.1186/s13019-023-02458-0 PMC10664312

[51] Yang Y, Li L, Hu H, Zhou C, Huang Q, Zhao J, et al. A nomogram integrating the clinical and CT imaging characteristics for assessing spread through air spaces in clinical stage IA lung adenocarcinoma. Front Immunol. 2025;16:1519766. 40292286 10.3389/fimmu.2025.1519766 PMC12021840

