# Supplementary figures and images for: CT feature-based nomogram for predicting tumor spread through air spaces in stage IA lung adenocarcinoma
Source: Cancer Imaging. 2025 Jun 11;25:72. doi: 10.1186/s40644-025-00893-x (PMC12160125; doi:10.1186/s40644-025-00893-x)

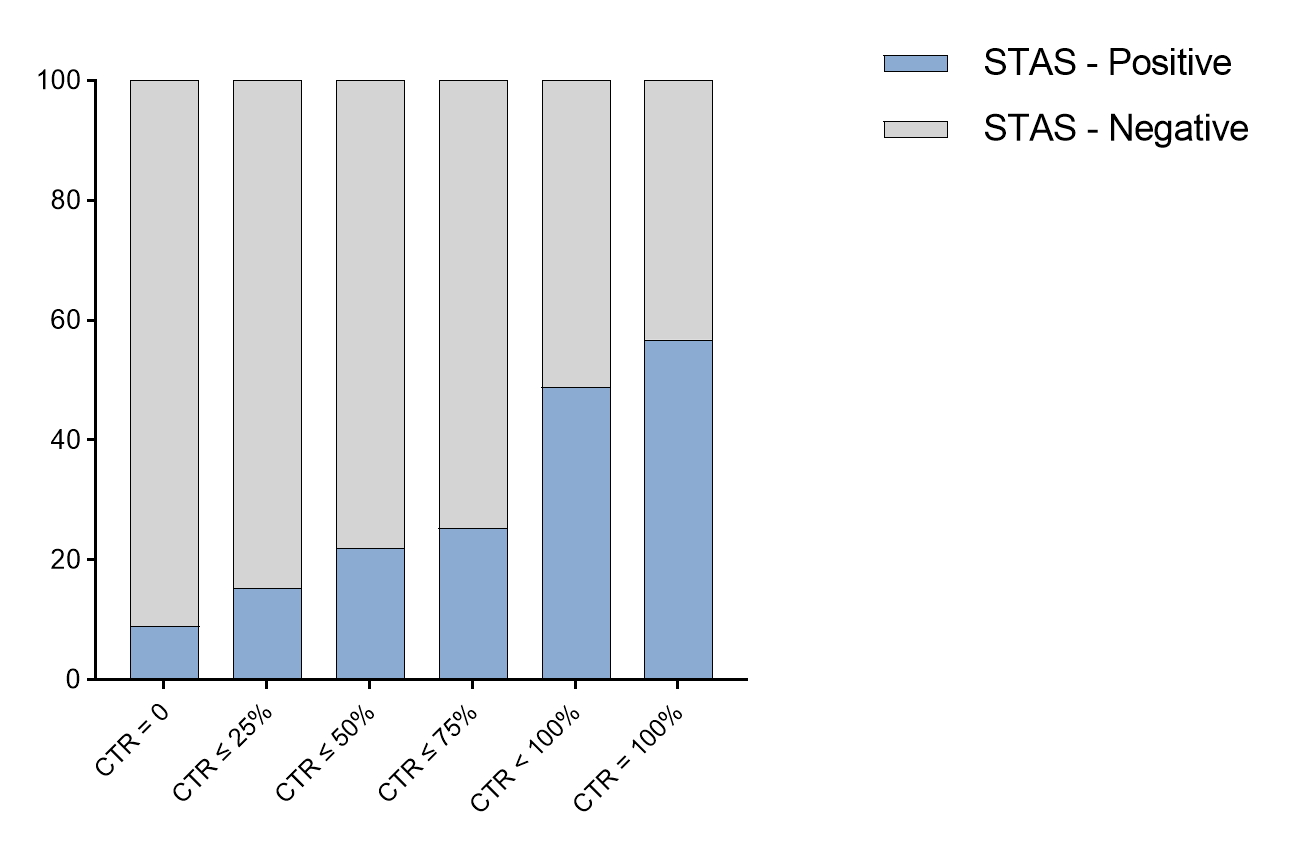

Supplement: Supplementary file 2 — Supplementary Material 2 [file 40644_2025_893_MOESM2_ESM.tif]
